# Contemplating cannabis? The complex relationship between cannabinoids and hepatic metabolism resulting in the potential for drug-drug interactions

**DOI:** 10.3389/fpsyt.2022.1055481

**Published:** 2023-01-10

**Authors:** Rosemary T. Smith, Staci A. Gruber

**Affiliations:** ^1^Marijuana Investigations for Neuroscientific Discovery (MIND) Program, McLean Imaging Center, McLean Hospital, Belmont, MA, United States; ^2^Department of Psychiatry, Harvard Medical School, Boston, MA, United States

**Keywords:** medical cannabis, cannabidiol (CBD), drug-drug interaction (DDI), CYP450, hepatic metabolism

## Abstract

The majority of states have fully legalized the use of medical cannabis (MC), and nearly all other states allow limited access to cannabidiol (CBD), a non-intoxicating constituent of cannabis often touted for a range of therapeutic indications. Further, the Agricultural Improvement Act of 2018 legalized hemp-derived products in all 50 states; typically high in CBD, these products are derived from cannabis varieties containing ≤0.3% delta-9-tetrahydrocannabinol (THC) by weight. The recent “green rush” has resulted in a striking increase in cannabis use among patients and consumers who often use a wide variety of novel product types, each with a unique blend of cannabinoid constituents. Importantly, however, several cannabinoids have the potential to cause drug-drug interactions (DDI) with other medications, primarily due to their involvement with the hepatic cytochrome P450 (CYP450) system. This article examines the potential for individual cannabinoids, particularly CBD, to interact with the hepatic metabolic system, which is concerning given its involvement in the metabolism of commonly-prescribed medications. CBD and other cannabinoids are metabolized extensively by the CYP450 system, and also inhibit many of these enzymes, potentially leading to variable serum levels of other medications, as well as variable levels of cannabinoids when other medications modify the system. As access and interest in cannabinoid-based products continues to increase, critical questions remain unanswered regarding their safety. The complex relationship between cannabinoids and the hepatic metabolic system, including common potential DDI resulting from cannabinoid exposure, are explored along with the clinical significance of these potential interactions and monitoring or mitigation strategies.

## 1. Introduction

*Cannabis sativa* is comprised of over 400 constituents, including more than 100 phytocannabinoids, many of which are known to have effects in the human body and demonstrate therapeutic potential (1). Until recently, despite widespread cannabis use, little research had elucidated the effects of cannabinoids on various biological processes. Two primary cannabinoids found in the plant are delta-9-tetrahydrocannabinol (THC), the most abundant cannabinoid and the primary intoxicating constituent, which has also demonstrated therapeutic benefits as an anti-emetic for chemotherapy-induced nausea and vomiting, pain, and muscle spasticity (2); and cannabidiol (CBD), often the second most abundant cannabinoid which is non-intoxicating and has been touted as therapeutic for a range of indications, including seizure disorders, anxiety, pain, and inflammation (3). In addition to THC and CBD, dozens of “minor” cannabinoids are found in the plant which are also often present in cannabis products, including cannabigerol (CBG), cannabichromene (CBC), cannabinol (CBN), cannabidivarin (CBDV), tetrahydrocannabivarin (THCV), and the acid forms of THC and CBD (THCA and CBDA), among others. While little work thus far has focused on minor cannabinoids, studies have shown that these compounds have a variety of biological effects (1, 4), and the presence of minor cannabinoids in medical cannabis (MC) products is increasing (5–7) as consumers and patients become aware of their potential utility.

Medical cannabis use has increased dramatically in recent years; in the U.S., almost all states have passed legislation allowing the use of MC or CBD-containing products. The rapid legalization of MC has coincided with a significant increase in MC use; state registry data indicates a 4.5 fold increase in registered MC patients from 2016 to 2020 (8), and use among older adults has increased particularly rapidly (9). As of November 2022, 37 states plus the District of Columbia (D.C.) have fully legalized the use of MC; in addition, 21 states plus D.C. have legalized adult or recreational use of cannabis (10, 11). Additionally, the Agricultural Improvement Act of 2018 (colloquially known as the “Farm Bill”) legalized hemp-derived products containing <0.3% THC by weight (12). While a synthetic form (dronabinol) and an analog (nabilone) of THC were approved by the U.S. Food and Drug Administration (FDA) in 1985 for treating chemotherapy-related nausea and vomiting, in 2018, the FDA approved Epidiolex, a plant-derived, purified CBD isolate for treatment-resistant, pediatric-onset seizure disorders. Epidiolex is the first (and only, to date) FDA-approved, cannabis-derived medication available in the U.S., while Sativex, a plant-derived 1:1 CBD:THC buccal spray, is available in several other countries (13). The convergence of legalization of cannabis and hemp, along with the more recent approval and availability of Epidiolex and Sativex, has resulted in a rapid increase in cannabinoid-based products available for purchase in dispensaries, through online retailers, and by prescription.

While the use of cannabinoid-containing products has increased significantly, relatively little work has focused on assessing potential drug-drug interactions (DDI) between cannabinoids and conventional medications. DDI can result in variable serum levels of substrates, leading to unexpected side effects, stronger drug effects than intended, or incomplete symptom relief due to lower efficacy (14). Several studies have demonstrated that cannabinoids interact with the cytochrome P450 (CYP450) enzyme system, the hepatic system responsible for the metabolism of most common medications, and the second phase of metabolism which further processes compounds for excretion (15–21). As these pathways are commonly implicated in DDI, increased cannabinoid use results in a major public health concern regarding potential DDI that has yet to be addressed. This article will provide a brief overview of the hepatic metabolic process and discuss potential areas of concern for interactions with cannabinoids (particularly CBD), the clinical significance of these interactions, and potential monitoring or mitigation strategies to minimize interactions which may help address public health concerns regarding DDI.

## 2. Hepatic metabolism: An overview

### 2.1. Phase I: CYP450 system

Drug metabolism primarily occurs in the liver, with secondary metabolism occurring at other sites including the intestines, kidneys, blood plasma, and lungs (22). Phase I of metabolism involves hydrolysis, reduction, and oxidation (the most common type of metabolism) (22), resulting in a metabolite that is commonly still active (23). Two additional phases of metabolism often occur; Phase II reactions create inactive compounds with increased polarity, often *via* glucuronidation, that are water-soluble and thus able to be excreted, while Phase III (which is uncommon and not discussed here in detail) further metabolizes Phase II compounds to allow for excretion (23). Together, Phase I metabolism *via* the CYP450 system and Phase II glucuronidation account for the metabolism of over 90% of conventional medications (18).

The CYP450 system is the major hepatic metabolic enzyme system that catalyzes Phase I reactions and is involved in the metabolism of the majority of common medications (24, 25). CYP450 is a hemeprotein superfamily comprised of over 50 enzymes/pathways (22) named with a family number (e.g., CYP1) and a subfamily letter (e.g., CYP1A), along with another number to determine the specific isoform or enzyme (e.g., CYP1A1) (22). Importantly, these enzymes do not typically work in isolation; multiple enzymes are often involved in the metabolism of a single medication or substrate.

In a report describing the characterization and distribution of CYP450 enzymes, Zanger and Schwab (24) noted that the enzymes most often associated with metabolizing typical medications were CYP3A4/5 (metabolizing > 30%); CYP2D6 (metabolizing > 20%); CYP2C9 (metabolizing > 13%); and CYP1A2 (metabolizing ∼9%). Other research has reported similar findings, confirming the critical role these enzymes play in metabolizing the majority of “most often prescribed” medications (26). Multiple factors impact functionality of each CYP enzyme, including polymorphisms, age, inflammation, illnesses/disease, and sex (24); variability in enzyme function over time may lead to fluctuations in metabolism within the same person, as well as inconsistent and variable levels of metabolism when compared to other individuals.

Modification of the CYP450 system by exogenous substances can alter metabolism of other substrates in two primary ways–inhibition and induction. Inhibition of CYP450 enzymatic activity is primarily accomplished by competitive binding; by occupying the enzyme’s active binding site, other substrates are displaced and are unable to be metabolized (27). The other main inhibitory method is non-competitive inhibition, in which the inhibitor binds to a different (allosteric) binding site than the substrate, changing the enzyme’s shape or function such that the substrate’s binding site is no longer available (27). Inhibition has the potential to result in incomplete metabolism and increased serum levels of concomitant medications, potentially leading to adverse events (28). Several medications are recognized as CYP450 inhibitors, including omeprazole, erythromycin, fluvoxamine, fluoxetine, haloperidol, ritonavir, and some antifungals including ketoconazole and fluconazole (27–29).

Induction is the second method by which exogenous substances typically modify the CYP450 system. Inducers activate a CYP enzyme, leading to increased enzymatic activity, which results in decreased bioavailability and increased clearance of certain medications (28). This is typically accomplished by activation of transcription factors resulting in increased expression of CYP enzymes (29). Many medications have been identified as CYP450 inducers, including carbamazepine, ethinyl estradiol, phenobarbital, dicloxacillin, and others [see Hakkola et al. for review (29)].

It is important to note that many medications with a narrow therapeutic index (TI), the ratio between a drug’s toxicity and effectiveness, rely on metabolism by the CYP450 system. Common medications with narrow TIs include anticoagulants, beta blockers, antidepressants, and antipsychotic medications. The enzyme CYP2C9 is particularly important, given many of its substrates include those with a narrow TI (24). Disruption of enzymatic activity may result in clinically significant changes in serum levels of these drugs with a narrow TI, leading to inadequate symptom relief or even adverse events.

### 2.2. Phase II metabolism

Phase II metabolism involves adding hydrophilic groups to the substrate or its metabolites to create water-soluble products for excretion (23). It involves multiple mechanisms, including methylation, acetylation, conjugation with amino acids or glutathione, or sulfation, but primarily involves glucuronidation using uridine 5′-diphosphoglucuronosyltransferase (UGT) enzymes, which link glucuronic acid to the substrate in order to increase polarity (13). A variety of UGT enzymes are involved in this process, and typically multiple UGTs are involved in glucuronidation of a single compound. Four broad families of UGT enzymes are involved in human drug metabolism: UGT1, UGT2, UGT3, and UGT8 (30).

Many common medications rely on activity of these enzymes, including over-the-counter (OTC) products like ibuprofen and other non-steroidal anti-inflammatory drugs (mainly relying on the UGT1A and 2B subfamilies), acetaminophen (primarily glucuronidated by the UGT1A subfamily), and prescription drugs including valproic acid, sorafenib, and propofol (13). Given that common OTC medications and prescription medications rely on glucuronidation, significant DDI could occur for many individuals.

## 3. Cannabinoid involvement with hepatic metabolic pathways

Medical cannabis and cannabinoids are available across a range of product types with many possible modes of use or routes of administration, resulting in variable impact with regard to metabolism. For example, inhalation (smoking or vaping) predominantly avoids first-pass metabolism (31) and is associated with a very rapid onset of action and relatively limited concern regarding DDI (15). Conversely, ingestion introduces cannabinoids through the gastrointestinal tract where they are processed, absorbed into the bloodstream, and travel to the liver where they undergo first-pass metabolism, resulting in a more delayed onset of action and raising significant concern regarding DDI (32). Cannabinoid metabolism may be impacted by medications that interact with hepatic metabolic pathways, potentially leading to greater side effects or unintended effects (such as intoxication), as well as directly impacting the metabolism of other substances relying on hepatic metabolism. Given the increasing availability and variety of cannabis and cannabinoid products, it is imperative to understand the potential interactions for both major and minor cannabinoids.

### 3.1. Cannabidiol (CBD)

Cannabidiol (CBD) has become increasingly popular given its potential therapeutic benefits without risk of intoxication. Given CBD-based products are typically used as edibles, capsules, or sublingual solutions/oils, and since CBD has been identified as the cannabinoid exhibiting the strongest interactions with the CYP450 system (21), CBD poses considerable risk for DDI.

Cannabidiol is metabolized extensively by the CYP450 system (13, 15, 17, 18), primarily by hydroxylation (21); while not all research agrees on the specific enzymes involved in CBD metabolism, it is clear that many are implicated. In addition to the CYP2C9, CYP2C19, CYP3A4, UGT1A9, and UGT2B7 enzymes involved in general cannabinoid metabolism (18), CYP2C8, CYP1A2, and CYP2B6 are also potentially implicated in CBD metabolism; several additional studies suggest that CYP2C9, CYP2C19, and CYP3A4 likely play the greatest role in metabolism of CBD (21, 33, 34).

Cannabidiol also modifies CYP450 enzyme function as an inhibitor and inducer. Several studies indicate that CBD inhibits CYP450 enzymes, typically due to competitive inhibition. Specifically, *in vitro* and *in vivo* studies have demonstrated that CYP2C9, CYP2C19, CYP2D6, and CYP3A enzymes are inhibited by CBD; CYP1A2, CYP2B6, and CYP2C8 may also exhibit reduced function after administration of CBD (15, 17, 18, 33). UGT1A9 and UGT2B7 are potentially inhibited by CBD administration as well (18, 35), indicating that not only is Phase I impacted, but Phase II inhibition is also possible. Additionally, CBD may modify the CYP450 system through induction; CBD may induce CYP1A2, CYP2B6, and CYP3A4 (20, 36). Other inhibitors or inducers of the CYP450 system may also affect the bioavailability of CBD, either increasing or decreasing serum levels, depending on the pathways implicated.

Investigations have only more recently begun to examine the potential clinical significance of interactions precipitated by CBD co-administration with other medications. While only a few studies have examined these effects, these investigations are especially useful in determining whether clinically meaningful interactions may affect the bioavailability of either CBD or concomitant medications. Bansal et al. (15, 16) precipitated interactions in a human liver microsome model and determined that strong interactions likely occur with high-dose oral CBD (700 mg) and CYP3A substrates, followed by moderate interactions with CYP1A2, CYP2B6, CYP2C8, CYP2C9, CYP2C19, and CYP2D6 substrates. In a clinical study, Gaston et al. (37) assessed serum levels of antiepileptic drugs following titration from 5 to 50 mg/kg/day of CBD in patients with epilepsy. Increasing CBD doses were associated with changes in serum levels of clobazam, rufinamide, topiramate, zonisamide, and eslicarbazepine, although levels remained within the acceptable serum range for each drug (37). Finally, in a recent clinical trial, Anderson et al. (38) examined CBD’s impact on serum levels of several medications used to treat anxiety disorders (fluoxetine, sertraline, citalopram, escitalopram, and mirtazapine), finding that common doses of CBD-containing products (200–800 mg/day) resulted in significantly increased citalopram serum levels in patients taking citalopram or escitalopram. The full clinical significance of these alterations is yet to be explored.

In addition to the risk of DDI, many medications have the potential to cause liver damage; pre-existing liver disease can significantly impact drug metabolism, resulting in substantial DDI (39). Liver function tests (LFTs) are a common way to monitor liver health and provide an important indicator of hepatic disease. Clinical trials of Epidiolex reported elevated LFTs in some individuals, which increased as the daily dose of Epidiolex increased; further, co-administration of Epidiolex with valproate and/or clobazam resulted in a significantly higher risk of elevated LFTs (3, 36). It is possible that these LFT elevations are clinically significant and have the potential to be serious; further investigation is warranted regarding the impact of CBD on LFTs with and without concomitant medication administration. Importantly, however, the prescribed daily dose of Epidiolex typically ranges from 5 to 20 mg/kg/day, which is significantly higher than typical doses of full- or broad-spectrum CBD products proliferating in the marketplace, raising the question of whether lower-dose CBD products are less concerning.

### 3.2. Delta-9-tetrahydrocannabinol (THC)

Considered the most abundant cannabinoid in the plant, and often sought by both recreational consumers and medical patients, delta-9-tetrahydrocannabinol (THC) is also likely to impact metabolic pathways, particularly the CYP450 system, causing potential DDIs. In their review, Kocis and Vrana (18) reported that CYP2C9, CYP2C19, CYP3A4, and UGT1A9 and UGT2B7 are the primary enzymes involved in cannabinoid metabolism, including THC. Several studies have demonstrated that THC may act as an inhibitor of CYP1A2, CYP2B6, CYP2C9, CYP2C19, CYP2D6, CYP2E1, and CYP2J2 (17, 19, 20). THC may also induce CYP1A1 and CYP2C9 (20). While many edible THC products are available in the marketplace, a significant number of THC-containing products are designed to be inhaled (i.e., smoked and vaped), which bypass first-pass metabolism in the liver, potentially limiting concerns related to DDI for at least some products (15).

### 3.3. Minor cannabinoids

Despite increasing presence in commercially-available products, relatively little research has focused on the impact of “minor” cannabinoids, which are less abundant in the plant than THC and CBD, and include CBG, CBC, CBN, THCV, CBDV, CBDA, and THCA. Although many “minors” are often only present in trace amounts in the plant and were historically present in very small amounts in cannabis and cannabinoid-based products, recent interest in their potential clinical benefit has resulted in products focused on delivering isolated minor cannabinoids (e.g., CBG and CBN) as well as combination products containing multiple cannabinoids. Cannabinol (CBN) is the most commonly studied minor cannabinoid, which has demonstrated inhibition of CYP1A1, CYP2B6, CYP2C9, and CYP2E1 (17, 19, 40, 41). Recently, Doohan et al. (17) evaluated the inhibitory potential of cannabinoids including 10 minor cannabinoids (THCA, THCV, THCVA, CBDA, CBDV, CBDVA, CBN, CBC, CBG, and CBGA) against CYP2B6, CYP2C9, CYP2C19, CYP2D6, and CYP3A4 *in vitro*. All minor cannabinoids except CBN inhibited CYP2C9, and most (CBDV, CBDVA, CBG, CBN, THCV, and THCVA) inhibited or partially inhibited CYP2C19. It is not clear from these *in vitro* studies whether minor cannabinoids inhibit the CYP450 system in a clinically meaningful way. Given their growing popularity and the increasing number of novel products in the marketplace containing considerable amounts of these constituents, additional research is needed to determine the likelihood of DDI related to minor cannabinoids.

## 4. Future directions

As access to MC products, particularly high CBD-containing products, continues to expand, critical questions remain unanswered regarding their safety. Although few studies have assessed the clinical significance of common DDI related to CBD exposure, evidence suggests moderate to strong interaction risks between CBD and drugs metabolized by a variety of CYP450 enzymes (15, 16), indicating that *interactions are likely at clinically-relevant doses of CBD*. Future studies are needed to fully evaluate the potential for cannabinoids to cause DDI; *in vivo* studies and human pharmacokinetic/pharmacodynamic (PK/PD) studies involving co-administration of multiple medications with cannabinoids will be particularly valuable in determining the clinical significance of any interactions. As DDI are also more likely with drugs with a narrow TI (17, 18, 22), additional co-administration studies are warranted, particularly for CYP2C9 substrates with a narrow TI (17). In addition, studies are necessary to assess whether DDI that result in changes in bioavailability actually lead to adverse outcomes in various clinical populations.

To date, potential mitigation strategies have not been studied. It is unlikely that an offset between administration of cannabinoids and concomitant medications of concern would completely address the issue, given the extremely long half-life of certain cannabinoids, which are lipophilic and remain detectable for days to weeks after use (42, 43); however, this should be evaluated directly. Monitoring strategies, including serial blood draws assessing serum levels of concomitant medications, and monitoring LFTs to avoid potential hepatic damage, could be utilized to minimize potential negative outcomes. Importantly, as the majority of older adults take medications involving the CYP450 system (44), this group is particularly important to monitor upon initiation of cannabinoid use. Consumers and health care providers alike should be informed regarding the potential for DDI when considering cannabis and cannabinoid use, and efforts should be made to eliminate or limit potential risk and harm.

## 5. Conclusion

The proliferation of medical cannabis and hemp-derived products has resulted in thousands of commercially available cannabinoid-based options in the marketplace. Many consider cannabis and cannabinoid-based products relatively harmless, especially those high in CBD which is non-intoxicating and often touted for its medical benefits. Unfortunately, concerns regarding the potential safety issues associated with their use in conjunction with other medications are often overlooked. While there is great promise in the use of cannabis and cannabinoid-based products for a range of conditions, researchers and healthcare providers should be aware of the potential for significant DDI and should counsel their patients regarding potential interactions whenever the use of cannabinoid-based products is disclosed or considered. As cannabis and cannabinoid use increases, particularly vulnerable groups (e.g., older adults) should understand the potential risks associated with using these products with concomitant medications.

## Data availability statement

The original contributions presented in this study are included in the article/supplementary material, further inquiries can be directed to the corresponding author.

## Author contributions

RS and SG designed and conceptualized the manuscript, responsible for writing and editing, and accountable for the full content of the manuscript. Both authors contributed to the article and approved the submitted version.

## References

[1] Rock E Parker L. Constituents of cannabis sativa. *Adv Exp Med Biol.* (2021). 1264:1–13. 10.1007/978-3-030-57369-0_1 33332000

[2] National Academies of Sciences, Engineering, and Medicine. *The health effects of cannabis and cannabinoids: the current state of evidence and recommendations for research.* Washington, DC: National Academies of Sciences, Engineering, and Medicine (2017). 28182367

[3] White C. A review of human studies assessing cannabidiol’s (CBD) therapeutic actions and potential. *J Clin Pharmacol.* (2019) 59:923–34. 10.1002/jcph.1387 30730563

[4] Walsh K McKinney A Holmes A. Minor cannabinoids: biosynthesis, molecular pharmacology and potential therapeutic uses. *Front Pharmacol.* (2021) 12:777804. 10.3389/fphar.2021.777804 PMC8669157 34916950

[5] Businesswire. *United States Minor Cannabinoids (CBG, CBC, CBN, THCV, CBGA) Markets Report 2021-2028 - Research And Markets.com*. (2022). Available online at: https://www.businesswire.com/news/home/20220110005551/en/United-States-Minor-Cannabinoids-CBG-CBC-CBN-THCV-CBGA-Markets-Report-2021-2028—ResearchAndMarkets.com (accessed September 27, 2022).

[6] BDSA. *CBD and CBD Sales See Rapid Growth as CBD Sales Slow in Cannabis Markets.* (2022). Available online at: https://bdsa.com/cbn-and-cbg-sales-see-rapid-growth (accessed September 27, 2022).

[7] Basen R. *Beyond CBD, THC: ‘Minor’ Cannabinoids Flood Market – Lack of Datais Concerning, Even for Practitioners who Believe in Medical Cannabis: Medpage Today*. (2021). Available online at: https://www.medpagetoday.com/special-reports/exclusives/91904 (accessed September 27, 2022).

[8] Boehnke K Dean O Haffajee R Hosanagar A. Trends in registration for medical cannabis and reasons for use from 2016 to 2020 : an observational study. *Ann Intern Med.* (2022) 175:945–51. 10.7326/M22-0217 35696691 PMC10233658

[9] Han B Palamar J. Trends in cannabis use among older adults in the united states, 2015-2018. *JAMA Intern Med.* (2020) 180:609–11. 10.1001/jamainternmed.2019.7517 32091531 PMC7042817

[10] ProCon.org. *State-by-State Medical Marijuana Laws.* (2022). Available online at: https://medicalmarijuana.procon.org/legal-medical-marijuana-states-and-dc (accessed September 27, 2022).

[11] ProCon.org. *State-by-State Recreational Marijuana Laws.* (2022). Available online at: https://marijuana.procon.org/legal-recreational-marijuana-states-and-dc (accessed September 27, 2022).

[12] U.S. Department of Agriculture. *Agriculture Improvement Act of 2018: Highlights and Implications.* (2022). Available online at: https://www.ers.usda.gov/agriculture-improvement-act-of-2018-highlights-and-implications (accessed December 27, 2022).

[13] Brown J Winterstein A. Potential adverse drug events and drug-drug interactions with medical and consumer cannabidiol (CBD) use. *J Clin Med.* (2019) 8:989. 10.3390/jcm8070989 31288397 PMC6678684

[14] Cascorbi I. Drug interactions–principles, examples and clinical consequences. *Dtsch Arztebl Int.* (2012) 109:546–55. 10.3238/arztebl.2012.0546 23152742 PMC3444856

[15] Bansal S Maharao N Paine M Unadkat J. Predicting the potential for cannabinoids to precipitate pharmacokinetic drug interactions via reversible inhibition or inactivation of major cytochromes P450. *Drug Metab Dispos.* (2020) 48:1008–17. 10.1124/dmd.120.000073 32587099 PMC7543485

[16] Bansal S Paine M Unadkat J. Comprehensive predictions of cytochrome P450 (P450)-mediated in vivo cannabinoid-drug interactions based on reversible and time-dependent p450 inhibition in human liver microsomes. *Drug Metab Dispos.* (2022) 50:351–60. 10.1124/dmd.121.000734 35115300 PMC11022902

[17] Doohan P Oldfield L Arnold J Anderson L. Cannabinoid interactions with cytochrome p450 drug metabolism: a full-spectrum characterization. *AAPS J.* (2021) 23:91. 10.1208/s12248-021-00616-7 34181150

[18] Kocis P Vrana K. Delta-9-tetrahydrocannabinol and cannabidiol drug-drug interactions. *Med Cannabis Cannabinoids.* (2020) 3:61–73. 10.1159/000507998 34676340 PMC8489344

[19] Nasrin S Watson C Perez-Paramo Y Lazarus P. Cannabinoid metabolites as inhibitors of major hepatic CYP450 enzymes, with implications for cannabis-drug interactions. *Drug Metab Dispos.* (2021) 49:1070–80. 10.1124/dmd.121.000442 34493602 PMC11022895

[20] Qian Y Gurley B Markowitz J. The potential for pharmacokinetic interactions between cannabis products and conventional medications. *J Clin Psychopharmacol.* (2019) 39:462–71. 10.1097/JCP.0000000000001089 31433338

[21] Zendulka O Dovrtelova G Noskova K Turjap M Sulcova A Hanus L Cannabinoids and cytochrome P450 interactions. *Curr Drug Metab.* (2016) 17:206–26. 10.2174/1389200217666151210142051 26651971

[22] McDonnell A Dang C. Basic review of the cytochrome p450 system. *J Adv Pract Oncol.* (2013) 4:263–8. 10.6004/jadpro.2013.4.4.7 25032007 PMC4093435

[23] Phang-Lyn S Llerena V. *Biochemistry, Biotransformation*. Treasure Island, FL: StatPearls Publishing (2022). 31335073

[24] Zanger U Schwab M. Cytochrome P450 enzymes in drug metabolism: regulation of gene expression, enzyme activities, and impact of genetic variation. *Pharmacol Ther.* (2013) 138:103–41. 10.1016/j.pharmthera.2012.12.007 23333322

[25] Lynch T Price A. The effect of cytochrome P450 metabolism on drug response, interactions, and adverse effects. *Am Family Phys.* (2007) 76:391–6. 17708140

[26] Prakash C Zuniga B Song C Jiang S Cropper J Park S Nuclear receptors in drug metabolism, drug response and drug interactions. *Nucl Receptor Res.* (2015) 2:101178. 10.11131/2015/101178 27478824 PMC4963026

[27] Deodhar M Al Rihani S Arwood M Darakjian L Dow P Turgeon J Mechanisms of CYP450 inhibition: understanding drug-drug interactions due to mechanism-based inhibition in clinical practice. *Pharmaceutics.* (2020) 12:846. 10.3390/pharmaceutics12090846 32899642 PMC7557591

[28] Ogu C Maxa J. Drug interactions due to cytochrome P450. *BUMC Proc.* (2000) 13:421–3. 10.1080/08998280.2000.11927719 16389357 PMC1312247

[29] Hakkola J Hukkanen J Turpeinen M Pelkonen O. Inhibition and induction of CYP enzymes in humans: an update. *Arch Toxicol.* (2020) 94:3671–722. 10.1007/s00204-020-02936-7 33111191 PMC7603454

[30] Jančová P Šiller M. Phase II drug metabolism.In: Paxton J editor. Topics on Drug Metabolism. (London: InTech) (2012). 10.5772/29996

[31] Lucas C Galettis P Schneider J. The pharmacokinetics and the pharmacodynamics of cannabinoids. *Br J Clin Pharmacol.* (2018) 84:2477–82. 10.1111/bcp.13710 30001569 PMC6177698

[32] Kim J De Jesus O. *Medication Routes of Administration.* TreasureIsland, FL: StatPearls Publishing (2022). 33760436

[33] Balachandran P Elsohly M Hill K. Cannabidiol interactions with medications, illicit substances, and alcohol: a comprehensive review. *J Gen Intern Med.* (2021) 36:2074–84. 10.1007/s11606-020-06504-8 33515191 PMC8298645

[34] Beers J Fu D Jackson K. Cytochrome P450-catalyzed metabolism of cannabidiol to the active metabolite 7-hydroxy-cannabidiol. *Drug Metab Dispos.* (2021) 49:882–91. 10.1124/dmd.120.000350 34330718 PMC11025033

[35] Al Saabi A Allorge D Sauvage F Tournel G Gaulier J Marquet P Involvement of UDP-glucuronosyltransferases UGT1A9 and UGT2B7 in ethanol glucuronidation, and interactions with common drugs of abuse. *Drug Metab Dispos.* (2013) 41:568–74. 10.1124/dmd.112.047878 23230132

[36] Jazz Pharmaceuticals. *Epidiolex (Cannabidiol) OralSolution: Full Prescribing Information.* (2018). Available online at: https://pp.jazzpharma.com/pi/epidiolex.en.USPI.pdf (accessed September 27, 2022).

[37] Gaston T Bebin E Cutter G Liu Y Szaflarski J Program U. Interactions between cannabidiol and commonly used antiepileptic drugs. *Epilepsia.* (2017) 58:1586–92. 10.1111/epi.13852 28782097

[38] Anderson L Doohan P Oldfield L Kevin R Arnold J Berger M Citalopram and cannabidiol: in vitro and in vivo evidence of pharmacokinetic interactions relevant to the treatment of anxiety disorders in young people. *J Clin Psychopharmacol.* (2021) 41:525–33. 10.1097/JCP.0000000000001427 34121064

[39] Palatini P De Martin S. Pharmacokinetic drug interactions in liver disease: an update. *World J Gastroenterol.* (2016) 22:1260–78. 10.3748/wjg.v22.i3.1260 26811663 PMC4716036

[40] Yamaori S Kushihara M Yamamoto I Watanabe K. Characterization of major phytocannabinoids, cannabidiol and cannabinol, as isoform-selective and potent inhibitors of human CYP1 enzymes. *Biochem Pharmacol.* (2010) 79:1691–8. 10.1016/j.bcp.2010.01.028 20117100

[41] Yamaori S Maeda C Yamamoto I Watanabe K. Differential inhibition of human cytochrome P450 2A6 and 2B6 by major phytocannabinoids. *Forensic Toxicol.* (2011) 29:117–24. 10.1007/s11419-011-0112-7

[42] Millar S Stone N Yates A O’Sullivan S. A systematic review on the pharmacokinetics of cannabidiol in humans. *Front Pharmacol.* (2018) 9:1365. 10.3389/fphar.2018.01365 30534073 PMC6275223

[43] Sharma P Murthy P Bharath M. Chemistry, metabolism, and toxicology of cannabis: clinical implications. *Iran J Psychiatry.* (2012) 7:149–56. 23408483 PMC3570572

[44] Cabrera M Dip R Furlan M Rodrigues S. Use of drugs that act on the cytochrome P450 system in the elderly. *Clinics.* (2009) 64:273–8. 10.1590/S1807-59322009000400002 19488582 PMC2694456

